# Public policies and their association with adolescent pregnancy in Southern Peru

**DOI:** 10.1186/s12978-025-02131-w

**Published:** 2025-09-30

**Authors:** Emilio Jose Medrano-Sánchez, Lizabeth Lourdes Alanya-Pereyra, Freddy Ochoa-Tataje

**Affiliations:** 1 Universidad San Ignacio de Loyola, Lima, Perú; 2 Universidad César Vallejo, Lima, Perú

**Keywords:** Adolescent pregnancy, Public policies, Sexual and reproductive health, Maternal morbidity, Adolescent pregnancy prevention

## Abstract

This study analyzed the association between public policies on adolescent pregnancy in a healthcare network in southern Peru, considering their alignment with Sustainable Development Goals (SDGs) 3, 4, and 5, which focus on health, education, and gender equality. The research was basic in nature, with a correlational quantitative approach and a non-experimental cross-sectional design. A structured survey with closed-ended Likert-scale questions was administered to 80 obstetrics professionals, selected through non-probabilistic convenience sampling. Instrument validity was established through expert judgment, and reliability was evaluated using Cronbach’s Alpha coefficient, obtaining a value of 0.83, which indicated high internal consistency. The results obtained via ordinal logistic regression revealed that public policies were significantly associated with adolescent pregnancy (*p* = 0.000), with a Nagelkerke determination coefficient of 0.737, indicating that approximately 73.7% of the variance in adolescent pregnancy was associated with the level of policy implementation. It was concluded that public policies were linked to lower perceived levels of adolescent pregnancy in the healthcare network under study, recommending the optimization of sexual and reproductive health strategies to strengthen their implementation.

**Supplementary Information:**

The online version contains supplementary material available at 10.1186/s12978-025-02131-w.

## Introduction

Adolescent pregnancy, defined as gestation occurring in young women between 10 and 19 years of age [1], constitutes a global public health challenge whose repercussions span social, economic, and health dimensions [2, 3]. In low- and middle-income countries, approximately 16 million births are reported each year in adolescents aged 15 to 19, with about one million occurring in those under 15. Such scenarios lead to medical complications including eclampsia, preterm birth, postpartum hemorrhages, and risk-related abortions [1, 4–6]. These issues are further exacerbated by contextual factors such as low educational attainment, economic insecurity, and sexual violence [7–10], which increase the likelihood of maternal and neonatal morbidity and mortality among adolescent mothers [11–13]. In addition, it has been shown that dysfunctional family environments, characterized by mental health issues, substance abuse, and domestic violence, further heighten adolescent vulnerability, necessitating culturally sensitive interventions and preventive approaches [14].

The consequences of early motherhood also include perpetuating the cycle of poverty and interrupting schooling, factors that notably limit adolescents’ job opportunities and personal development [15, 16]. Studies in Latin America and the Caribbean show that the region posts particularly high adolescent fertility rates, second only to sub-Saharan Africa, especially in rural areas and in socioeconomically disadvantaged populations [17, 18]. In Peru, although fluctuations have been observed in the pregnancy rate for adolescents aged 15 to 19, a concerning increase has emerged in pregnancies among girls between 10 and 14 years of age, also accompanied by a rise in sexual violence [18–21]. In regions such as Ica, the adolescent population is projected to grow, accompanied by an uptick in births among mothers under age 15, a situation underscoring the urgency of multisectoral interventions [18].

In light of this reality, various international and regional organizations, such as the Pan American Health Organization (PAHO), the Economic Commission for Latin America and the Caribbean (ECLAC), and the United Nations Population Fund (UNFPA), have proposed solutions centering on strengthening public policies in sexual and reproductive health, promoting comprehensive sex education, and ensuring universal access to contraceptive methods [22–25] These strategies align with the Sustainable Development Goals (SDGs), especially those that aim to reduce maternal mortality and foster health and well-being (SDG 3), ensure quality education (SDG 4), and achieve gender equality (SDG 5), since adolescent pregnancy poses an obstacle to progress in these areas [1, 26, 27]. Nonetheless, research suggests that many official initiatives remain symbolic, lacking in funding mechanisms and concrete procedures [28, 29]. In countries such as Brazil or South Africa, for example, limited access to youth-friendly services and hostile attitudes of healthcare staff continue to hinder the prevention of unwanted pregnancies [30, 31]. Likewise, in the United States, restrictive policies have been found to raise adolescent pregnancy rates, whereas expanding access to family planning methods lowers birth rates in this age group [32, 33].

From the perspective of public health policy management, authors such as [34] stress the importance of analyzing policy content, the context in which it is framed, the actors involved, and the processes of formulation and implementation. Meanwhile, Kingdon’s multiple-streams framework [35, 36] and the three-delays theory [37] illustrate how certain issues, such as adolescent pregnancy, gain priority on the political agenda when critical contextual circumstances, technically feasible solutions, and politically favorable environments converge. In Peru, the technical guidelines issued by the Ministry of Health emphasize the need to offer comprehensive and differentiated services for this population [38, 39]. However, the limited compliance with these norms at the local level signals the persistence of cultural, institutional, and resource-related barriers [40–42].

Within this analytical framework, the guiding research question sought to determine “What is the association between public policies on adolescent pregnancy in a healthcare network in southern Peru, 2024?” Specifically, the study examined the association of these policies on the following dimensions: early adolescent pregnancy (10 to 14 years), late adolescent pregnancy (15 to 19 years), maternal morbidity, and maternal mortality. Based on these objectives, the overarching goal was to determine the association of public policies on adolescent pregnancy in a healthcare network in southern Peru, 2024. The study’s specific aims included analyzing the association of public policies on early adolescent pregnancy, late adolescent pregnancy, maternal morbidity, and maternal mortality within the same healthcare network [10, 18].

Finally, grounded in the available evidence and in the gap between policy formulation and on-the-ground implementation, a general hypothesis was proposed, positing that public policies are associated with adolescent pregnancy in a healthcare network in southern Peru, 2024. Specific hypotheses were likewise set forth indicating that public policies also have an impact on early adolescent pregnancy, late adolescent pregnancy, maternal morbidity, and maternal mortality. These premises are crucial for guiding effective strategies that enhance education, prevention, and quality of care, thus contributing to the achievement of the SDGs and, ultimately, to the well-being of young women in Peru.

## Methodology

The research was basic in nature, aiming to expand scientific knowledge without prompting an immediate application [43]. Additionally, a correlational quantitative approach was adopted, as two or more variables were examined statistically based on a hypothesis proposing a possible association between variables [44–46]. The study employed a non-experimental design, given that no variables were manipulated, and the reality was observed in its natural state [47]. It was also cross-sectional, given that data were collected at a single point in time, simultaneously measuring both exposure and outcome [48].

In this context, the variable “Public Policies” was defined as the set of government actions and decisions aimed at addressing a problematic social situation [49]. To analyze its association with adolescent pregnancy, two operational dimensions were considered: sexual health, which included age of sexual initiation, the presence of sexual violence, and sexually transmitted infections; and reproductive health, encompassing access to family planning and differentiated obstetric care. Meanwhile, adolescent pregnancy was defined as one occurring between the ages of 10 and 19 [1, 50] and was operationalized through four dimensions: early adolescence (10 to 14 years), late adolescence (15 to 19 years) [51], maternal morbidity, and maternal mortality [37].

The study population comprised 38 obstetrician-gynecologists and 63 non-physician obstetricians employed in a healthcare network in southern Peru. Inclusion criteria required holding a specialty in gynecology and/or obstetrics, maintaining an active employment relationship, being in active practice during the study period, and voluntarily agreeing to participate. Consequently, professionals from other health specialties or those on leave during the data collection period were excluded. To ensure the availability and relevance of the participants for the study requirements, a non-probabilistic convenience sampling was applied [47], resulting in the inclusion of 80 professionals according to the sample size formula. Sampling was intentional rather than probabilistic; the investigators selected all obstetric professionals who were on duty during the single visit to each facility, prioritizing feasibility given the project’s limited budget and timeframe.

Information was gathered through a structured survey with closed-ended Likert-scale questions administered virtually. This instrument was designed in accordance with the indicators established in the consistency matrix and underwent expert judgment for validation, thereby ensuring its relevance. To verify reliability, Cronbach’s Alpha coefficient was calculated, obtaining a value of 0.83, indicating high internal consistency [52, 53]. The final instrument comprised 28 Likert-type items distributed in six domains. For the explanatory variable Public Policies there were two domains, sexual-health policy (6 items) and reproductive-health policy (4 items). The outcome variable Adolescent Pregnancy included four domains, early pregnancy (7 items), late pregnancy (7 items), maternal morbidity (2 items) and maternal mortality (2 items). A sample item for the sexual-health domain is “Public policies have reduced early sexual initiation to prevent adolescent pregnancy.” Complete item wording and the anonymized response dataset are provided in Supplementary File 1 and Supplementary File 2, respectively. Data were processed using SPSS software (version 29.0.2.0). Initially, the Kolmogorov-Smirnov test was applied, revealing a lack of normality in the distribution (*p* < 0.05).

About the study design and originality statement, this study is based on primary quantitative data collected between October 2024 and January 2025 from 80 reproductive-health professionals (79% of the 101 eligible staff) working in 12 facilities of the Southern Peru Health Network. Respondents completed a 28-item Likert-type questionnaire (1 = Strongly disagree … 5 = Strongly agree) covering two exposure domains, sexual-health and reproductive-health public policies, and four outcome domains related to adolescent pregnancy (early, late, morbidity and mortality). The questionnaire was content validated by five experts and showed good internal consistency (Cronbach’s α = 0.83 for the total scale). Full item wording and the anonymized raw dataset are provided as Supplementary File 1 and Supplementary File 2, respectively.

Based on this result, logistic regression was employed to analyze the association between the variables, complemented by descriptive statistics using frequencies and percentages.

The study was conducted under the ethical principles of scientific integrity, transparency, honesty, and objectivity, while also safeguarding participant autonomy, as each participant gave voluntary informed consent. Likewise, the necessary institutional permissions were secured, and the confidentiality of the collected information was ensured, with data used solely for academic purposes [49]. By upholding these ethical and methodological standards, the research provided an updated perspective on adolescent pregnancy and offered inputs for designing more effective sexual and reproductive health strategies and policies.

## Results

This chapter presents the findings obtained after processing the statistical information gathered through the survey administered to healthcare professionals. First, the general characteristics of the variables are described, including frequency distributions and percentages that outline the reality under study. This descriptive analysis provides an initial view of how public policies and adolescent pregnancy behave, as well as their associated dimensions (early, late, maternal morbidity, and maternal mortality). Subsequently, the inferential results based on ordinal logistic regression are introduced, demonstrating the statistically significant relationship between these policies and adolescent pregnancy. This deeper analysis allows for the verification of the hypotheses, highlighting the association of public policies in both preventing and managing pregnancy among those under 19 years of age. In this way, the descriptive results offer an introductory overview of the data collected, while the inferential findings provide quantitative support for understanding the magnitude and direction of the effects observed.

### Descriptive results

Table 1 shows how respondents rate the presence of public policies in relation to sexual and reproductive health. In the general “Public Policies” variable, the medium level (61.3%) predominates, followed by a 30.0% rating them as high and 8.8% considering them low. This distribution suggests that, although most professionals acknowledge the existence of guidelines and efforts aimed at preventing adolescent pregnancy, they still perceive these measures as insufficiently consistent or integrated to achieve an optimal level. The group that rates them as high (30.0%) implies there are facilities or areas where policy adherence is solid, potentially leading to better results in sexual education and family planning. However, the 8.8% that considers these policies to be at a low-level points to significant gaps, whether due to irregular implementation or shortcomings in resources, training, or inter-institutional coordination.

**Table 1:** Levels of public policies and their dimensions

Levels	Public Policies		Sexual Health		Reproductive Health	
	f	%	f	%	f	%
Low	7	8,8	15	18,8	9	11,3
Medium	49	61,3	42	52,5	48	60,0
High	24	30,0	23	28,8	23	28,8
Total	80	100,0	80	100,0	80	100,0

Within these specific dimensions, sexual health shows a higher low level (18.8%) than overall public policies (8.8%). This difference may indicate that, although general guidelines exist, their translation into sexual education programs, contraceptive availability, and sexual violence prevention is not always effective. The majority (52.5%) places it at a medium level, and 28.8% at a high level, revealing a relative advance in sexual health prevention and promotion; nevertheless, a significant proportion of professionals perceive deficiencies.

By contrast, reproductive health is rated at 60.0% medium and 28.8% high, while the low level (11.3%) is lower than that of sexual health (18.8%). This finding could point to greater consolidation of strategies linked to differentiated obstetric care and family planning compared to sexual education. However, the fact that most respondents rate it only as medium suggests that heightened emphasis is still required on supervision, resource allocation, ongoing staff training, and the development of reproductive health protocols.

Overall, Table 1 reflects a context in which public policies, while largely acknowledged at a medium or high level, are not viewed as fully effective by all professionals, particularly in matters concerning sexual health. This underscores the need for more focused and continuous interventions in the areas deemed deficient, in order to achieve a clearer and more sustainable reduction in adolescent pregnancy.

Table 2 focuses on the “Adolescent Pregnancy” variable and its four dimensions: early adolescent pregnancy (10–14 years), late adolescent pregnancy (15–19 years), maternal morbidity, and maternal mortality. A total of 57.5% of respondents rate adolescent pregnancy at a medium level, 35.0% at a high level, and only 7.5% at a low level. This distribution indicates that the issue is viewed as either significant or at least moderately frequent in clinical and healthcare practice, aligning with the increasing importance attributed to this topic in various health policies.

**Table 2:** Levels of adolescent pregnancy and its dimensions

Levels	Adolescent Pregnancy		Early		Late		Morbidity		Mortality	
	f	%	f	%	f	%	f	%	f	%
Low	6	7,5	6	7,5	6	7,5	6	7,5	6	7,5
Medium	46	57,5	47	58,8	48	60,0	49	61,3	49	61,3
High	28	35,0	27	33,8	26	32,5	25	31,3	25	31,3
Total	80	100,0	80	100,0	80	100,0	80	100,0	80	100,0

When examining the specific dimensions, both early (58.8% medium; 33.8% high) and late adolescent pregnancy (60.0% medium; 32.5% high) exhibit similar profiles, with a majority in the medium category and about one-third in the high category. This congruence underscores that, regardless of whether the pregnancy occurs at age 12 or 17, professionals view gestation in individuals under 19 as a recurring and concerning phenomenon. The low percentage in both dimensions (around 7.5%) indicates that few respondents consider adolescent pregnancy to have a minimal or negligible incidence, confirming its priority status within healthcare services.

Regarding maternal morbidity and mortality, medium levels (61.3% in each case) and high levels (31.3%) indicate a significant number of pregnant adolescents face complications during pregnancy, childbirth, or the postpartum period, some even at risk of death. Although the proportion at the low level is minimal (7.5%), its mere existence suggests the possibility of certain environments with a lower incidence of complications or better healthcare responses. Nonetheless, the overall prevalence of moderate and severe cases calls for continued vigilance, early detection of disorders, and specialized obstetric care to reduce the likelihood of fatal outcomes among adolescents.

In summary, Table 2 reveals that, in the perception of professionals, adolescent pregnancy is seen as an issue with a predominantly moderate incidence yet with a considerable proportion of serious cases. The fact that early and late pregnancies, as well as maternal morbidity and mortality, are clustered around medium and high levels underscores the need for more robust and coordinated interventions to safeguard adolescents’ health and life trajectory.

### Inferential results

#### Normality test

To evaluate the shape of the data distribution and determine the appropriateness of using parametric or non-parametric procedures, the Kolmogorov-Smirnov (K-S) test was applied to a sample of 80 participants (*n* > 50). Table 3 presents the corresponding results.

**Table 3:** Normality test

	Kolmogorov-Smirnov			
	Statistic	gl	Sig.	
Public policies	3,049	80	,000	
Adolescent pregnancy	2,934	80	,000	

As shown, both variables yielded significance (Sig.) values lower than 0.05, implying the null hypothesis of normality is rejected and indicating that the data do not follow a normal distribution. This finding is crucial, as the failure to meet this statistical assumption directs the choice of non-parametric tests for subsequent analysis. Consequently, in studying the relationship between public policies and adolescent pregnancy from an ordinal perspective, ordinal logistic regression was employed. This technique does not require normality and allows for appropriate modeling of the dependent variable “Adolescent Pregnancy” in terms of the different levels of the independent variable “Public Policies.”

#### Inferential analysis

Because the normality tests showed significance values below 0.05, non-parametric statistical methods were deemed necessary to test the general hypothesis, which stated that public policies were associated with adolescent pregnancy in the healthcare network in southern Peru.

In this regard, ordinal logistic regression was used to examine the relationship between the variable “Public Policies” and “Adolescent Pregnancy.” When comparing the initial model, which only included the intercept, with the final model, the former presented a value of −2 log likelihood of 96.730, while the latter reached 21.377, as shown in Table 4.

**Table 4:** Model evaluation report for the general hypothesis

Model	−2 log likelihood	Chi-sqare	df.	Sig.
Only intercept	96,730			
Final	21,377	75,353	2	,000

This statistically significant improvement was reflected in a Chi-square of 75.353, with a p value of 0.000, demonstrating that the inclusion of public policies provided considerable explanatory power in the behavior of adolescent pregnancy.

To quantify the model’s predictive capacity, several R^2^ measures were calculated, as shown in Table 5. Among them, Nagelkerke’s R^2^ stood out with a value of 0.737, indicating that approximately 73.7% of the variability in the presence or absence of adolescent pregnancy was related to the “Public Policies” variable.

**Table 5:** Determination coefficient for the general hypothesis

*R* ^2^	
Cox y Snell	,610
Nagelkerke	,737
McFadden	,535

Moreover, an examination of the model’s coefficients (Table 6) revealed that the values associated with low and medium levels of these policies were negative and statistically significant compared to the high level. Thus, when the scope or effectiveness of public policies diminished, adolescent pregnancy was more likely to be found at moderate or high levels. These findings confirmed the hypothesis and suggested that the continuity and strength of governmental guidelines play a determining role in reducing the risk of pregnancy among those under 19 in the healthcare network studied.

**Table 6:** Coefficients of public policies in adolescent pregnancy

		Estimation	Std. Error.	Wald	df	Sig.	95% Confidence Interval	
							Lower limit	Upper limit
Threshold	[v3 = 1]	−8,293	1,314	39,809	1	,000	−10,870	−5,717
	[v3 = 2]	−2,398	,739	10,542	1	,001	−3,846	-,950
Location	[v1 = 1]	−9,903	1,660	35,599	1	,000	−13,157	−6,650
	[v1 = 2]	−4,392	,859	26,115	1	,000	−6,077	−2,708
	[v1 = 3]	0^a^	.	.	0	.	.	.

An ordinal logistic regression analysis was conducted to verify the incidence of public policies on early adolescent pregnancy, in line with Specific Hypothesis 1, which posited a relationship between both variables within the healthcare network in southern Peru. The comparison between the intercept-only model and the final model appears in Table 7. The former recorded a −2 log likelihood of 91.695, whereas the latter was 21.716. This improvement, reflected in a Chi-square of 69.979 and a p value of 0.000, showed that the inclusion of public policies significantly explained the phenomenon of early adolescent pregnancy.

**Table 7:** Model evaluation report for specific hypothesis 1

Model	−2 log likelihood	Chi-square	df	Sig.
Only intercept	91,695			
Final	21,716	69,979	2	,000

To determine the magnitude of this explanation, R^2^ measures were computed, as shown in Table 8. The Cox & Snell indicator was 0.583, Nagelkerke 0.706, and McFadden 0.501. A Nagelkerke value of 0.706 indicated that around 70.6% of the variation in “early adolescent pregnancy” was related to the level of public policies, reinforcing the notion that this variable played a significant role in the likelihood of gestation occurring at very early ages.

**Table 8:** Determination coefficient specific for hypothesis 1

*R* ^2^	
Cox y Snell	,583
Nagelkerke	,706
McFadden	,501

Table 9 shows the coefficients that describe the effect of public policies on early adolescent pregnancy. Regarding the thresholds, negative estimates for [ddd1 = 1] and [ddd1 = 2] were statistically significant, indicating that moving from a lower to a higher level of early adolescent pregnancy was not random but was connected to the strength of the policies. Examining the “location” section, the coefficients related to the low and medium policy levels were likewise negative and statistically significant compared to the high level, demonstrating that as the presence or effectiveness of public policies weakened, the probability that adolescents would fall into higher categories of early pregnancy increased. These findings confirmed Specific Hypothesis 1 by showing that public policy consolidation and continuity were closely related to preventing and reducing the incidence of pregnancy in girls under 15 within the study area.

**Table 9:** Coefficients of public policies on early adolescent pregnancy

		Estimation	Std. Error	Wald	df	Sig.	95% Confidence Interval	
							Lower limit	Upper limit
Threshold	[ddd1 = 1]	−7,845	1,250	39,417	1	,000	−10,295	−5,396
	[ddd1 = 2]	−1,946	,617	9,942	1	,002	−3,156	-,736
Location	[v1 = 1]	−9,456	1,609	34,535	1	,000	−12,609	−6,302
	[v1 = 2]	−3,941	,758	27,052	1	,000	−5,426	−2,456
	[v1 = 3]	0^a^	.	.	0	.	.	.

Continuing the previous analyses, Specific Hypothesis 2, postulating that public policies are associated with late adolescent pregnancy in the healthcare network in southern Peru, was tested. Once again, ordinal logistic regression was carried out to examine how “Public Policies” affect the level of adolescent pregnancy in individuals aged 15 to 19. The comparison between the model including only the intercept and the final model is shown in Table 10. The former had a −2 log likelihood of 80.453, whereas the latter reached 21.902, indicating a Chi-square difference of 58.552 with a p value of 0.000. This outcome showed that adding public policies offered a significant explanation of late adolescent pregnancy.

**Table 10:** Model evaluation report for specific hypothesis 2

Model	−2 log likelihood	Chi-square	df	Sig.
Only intercept	80,453			
Final	21,902	58,552	2	,000

To assess predictive capacity, Cox & Snell, Nagelkerke, and McFadden measures were calculated, as shown in Table 11. Nagelkerke’s R^2^ reached 0.631, indicating that approximately 63.1% of the variability in late adolescent pregnancy was tied to the presence or effectiveness of public policies.

**Table 11:** Determination coefficient for specific hypothesis 2

*R* ^2^	
Cox y Snell	,519
Nagelkerke	,631
McFadden	,423

The coefficient analysis in Table 12 showed that thresholds [ddd2 = 1] and [ddd2 = 2], with values of −7.071 and − 1.336, respectively, were statistically significant, indicating that moving from one level of late adolescent pregnancy to another was not random but instead related to the impact of public policies. Additionally, the location coefficients for low and medium policy levels were negative (−8.681 and − 3.155, respectively) and significant compared to the high level, interpreted as an increased likelihood of higher late adolescent pregnancy levels when policies lacked robustness or were poorly implemented. These findings confirmed Specific Hypothesis 2, highlighting that the continuity and strength of government strategies aimed at adolescent reproductive health played a pivotal role in reducing pregnancy among those aged 15 to 19.

**Table 12:** Coefficients of public policies on late adolescent pregnancy

		Estimation	Std. Error	Wald	df	Sig.	95% Confidence Interval	
							Lower limit	Upper limit
Threshold	[ddd2 = 1]	−7,071	1,185	35,621	1	,000	−9,393	−4,749
	[ddd2 = 2]	−1,336	,503	7,063	1	,008	−2,321	-,351
Location	[v1 = 1]	−8,681	1,559	31,003	1	,000	−11,737	−5,626
	[v1 = 2]	−3,155	,649	23,594	1	,000	−4,428	−1,882
	[v1 = 3]	0^a^	.	.	0	.	.	.

To test Specific Hypothesis 3, positing that public policies impacted maternal morbidity in the healthcare network in southern Peru, ordinal logistic regression was once again carried out. According to Table 13, the intercept-only model had a −2 log likelihood value of 71.343, whereas the final model, including the “Public Policies” variable, showed a value of 21.946, representing a 49.397 change in Chi-square with a p value of 0.000. This result indicated that incorporating public policies contributed a statistically significant explanation for variations in maternal morbidity.

**Table 13:** Model evaluation report for specific hypothesis 3

Model	−2 log likelihood	Chi-square	df	Sig.
Only intercept	71,343			
Final	21,946	49,397	2	,000

Predictive capacity was measured through the R^2^ indicators detailed in Table 14, where Nagelkerke’s value reached 0.562, suggesting that around 56.2% of the variation in maternal morbidity was linked to the “Public Policies” variable.

**Table 14:** Determination coefficient for specific hypothesis 3

*R* ^2^	
Cox y Snell	,461
Nagelkerke	,562
McFadden	,360

The coefficient analysis in Table 15 showed that thresholds [ddd3 = 1] and [ddd3 = 2] were negative and statistically significant, indicating that transitions among different morbidity levels were not random but rather connected to how strong or weak public policies were. In the location section, low and medium policy levels also yielded negative and significant coefficients, indicating that as the reach or effectiveness of these policies decreased, the probability of maternal morbidity occupying higher categories increased. This confirmed Specific Hypothesis 3 by demonstrating that the consistency and continuity of government guidelines played a key role in preventing health complications during pregnancy, childbirth, or the postpartum period in the adolescent population.

**Table 15:** Coefficients of public policies in maternal morbidity

		Estimation	Std. Error	Wald	df	Sig.	95% Confidence Interval	
							Lower limit	Upper limit
Threshold	[ddd3 = 1]	−6,487	1,152	31,690	1	,000	−8,745	−4,228
	[ddd3 = 2]	-,889	,449	3,921	1	,048	−1,770	-,009
Location	[v1 = 1]	−8,097	1,535	27,841	1	,000	−11,104	−5,089
	[v1 = 2]	−2,553	,594	18,464	1	,000	−3,717	−1,388
	[v1 = 3]	0a	.	.	0	.	.	.

Using the same analytical methodology, ordinal logistic regression was employed to test Specific Hypothesis 4, which held that public policies are associated with maternal mortality in adolescents within the healthcare network in southern Peru. Table 16 compares the model that included only the intercept with the final model incorporating “Public Policies.” In the former, the − 2 log likelihood was 73.627, whereas the latter reached 21.345, signifying a 52.282 change in the Chi-square statistic, with a significance of 0.000. This revealed that the inclusion of public policies was relevant in explaining maternal mortality among adolescents.

**Table 16:** Model evaluation report for specific hypothesis 4

Model	−2 log likelihood	Chi-square	df	Sig.
Only intercept	73,627			
Final	21,345	52,282	2	,000

To estimate the model’s predictive capacity, the R^2^ values were reviewed (Table 17). Nagelkerke reached 0.578, indicating that roughly 57.8% of the variation in maternal mortality was linked to the level of public policy intervention.

**Table 17:** Determination coefficient for specific hypothesis 4

*R* ^2^	
Cox y Snell	,480
Nagelkerke	,578
McFadden	,369

Table 18 presents the regression coefficients, in which thresholds [ddd4 = 1] and [ddd4 = 2] were negative and statistically significant, indicating that moving from a lower to a higher category of maternal mortality did not occur by chance, but was linked to the solidity of public policies. Additionally, coefficients for low and medium policy levels showed negative, significant values compared to the high level, revealing that diminished strength or implementation of these policies increased the probability that maternal mortality in adolescents would be classified in higher tiers. These findings confirmed Specific Hypothesis 4 and underscored the importance of consistent and robust official directives to reduce fatal outcomes among pregnant adolescents.

**Table 18:** Coefficients of public policies in maternal mortality

		Estimation	Error típ.	Wald	df	Sig.	95% Confidence Interval	
							Lower limit	Upper limit
Threshold	[ddd4 = 1]	−6,656	1,165	32,668	1	,000	−8,938	−4,373
	[ddd4 = 2]	−1,337	,503	7,067	1	,008	−2,322	-,351
Location	[v1 = 1]	−8,266	1,544	28,680	1	,000	−11,292	−5,241
	[v1 = 2]	−2,727	,616	19,568	1	,000	−3,935	−1,519
	[v1 = 3]	0a	.	.	0	.	.	.

In summary, statistically significant associations were consistently identified across all analyzed dimensions of adolescent pregnancy, as detailed in Tables 6, 8, 10 and 12. These tables report logistic-regression coefficients (β), standard errors, p-values (all < 0.001) and Nagelkerke R² values ranging from 0.562 to 0.737, demonstrating strong explanatory power of the public-policy variables.

## Discussion

Regarding the general hypothesis, which posited that public policies were significantly associated with adolescent pregnancy in a healthcare network in southern Peru, the ordinal logistic regression results revealed statistically significant values, with a Chi-square change of 75.353 and a p value of 0.000 when comparing the base model against the final model. Likewise, Nagelkerke’s R^2^ reached 0.737, indicating that approximately 73.7% of the variance in adolescent pregnancy was explained by the presence or effectiveness of public policies. These findings confirmed the general hypothesis and suggested that government directives focusing on sexual and reproductive health played an important explanatory role in how adolescent pregnancy levels were distributed among the study population. Analyzing the regression coefficients showed that low and medium levels of public policies were associated with a higher likelihood of classifying adolescent pregnancy in more elevated categories, underscoring that strengthening and maintaining such policies may play a key role in lowering the likelihood of adolescent pregnancy within this age group.

These results align with the reflections of authors such as [28], who highlighted the need for policies on adolescent pregnancy to move beyond symbolic declarations, adopting concrete actions supported by established procedures and allocated budgets. They also accord with [29], who argued that deficient policy implementation leads to increased costs for health systems and greater risks for adolescents by failing to ensure effective access to contraceptive methods or sexual education. Meanwhile, these findings are consistent with [31], who noted that young people’s perception of difficulties in obtaining preventative methods, combined with providers’ attitudes, can hinder the effectiveness of any governmental strategy. Consequently, the evidence from this study regarding the importance of public policies supports the notion that a consistent and sustained regulatory framework can significantly lower adolescent pregnancy rates and potential complications.

In Specific Hypothesis 1, which suggested that public policies affected early adolescent pregnancy (ages 10 to 14), the final model showed a Chi-square change of 69.979 (*p* = 0.000). Nagelkerke’s R^2^ reached 0.706, indicating that 70.6% of the variation in early adolescent pregnancy was linked to the strength of public policies. Negative coefficients for both low and medium levels reinforced that weaker state actions resulted in younger adolescents becoming more prone to higher levels of pregnancy, as [30] also pointed out. Beyond emphasizing the need for comprehensive sex education, these authors highlighted the importance of having obstetric services prepared to care for adolescents whose socioeconomic conditions might increase pregnancy risk. Similarly [26], contended that designing targeted policies is essential to bridging the gap at very early ages, fully coinciding with the results obtained here. Nonetheless [31], questioned the behavior of certain healthcare professionals, implying that even if policies exist, their effectiveness in preventing early pregnancy could be undermined if providers are not trained or sensitized. This suggests that although an adequate normative framework may be in place, cultural factors and the healthcare system itself can dampen its impact.

In Specific Hypothesis 2, focused on the association between public policies in late adolescent pregnancy (ages 15 to 19), the study observed a Chi-square change of 58.552 (*p* = 0.000), and Nagelkerke’s R^2^ was 0.631, meaning 63.1% of the variance in late adolescent pregnancy was explained by the “Public Policies” variable. The coefficient analysis again yielded negative and significant results for low and medium levels of public policies, revealing that the probability of reaching high levels of late adolescent pregnancy increased when governmental directives were less present or effective.

This finding is consistent with [27], who emphasize that, during the latter half of adolescence, awareness of sexual rights and access to quality information exert a notable effect on pregnancy prevention. Simultaneously [31], underscored the importance of healthcare staff attitudes and the regulations that facilitate the provision of contraceptive methods without administrative barriers. It is worth noting that [33], after examining public policies in various U.S. states, found a significant correlation between expansive family planning policies and fewer pregnancies in this age group, thus corroborating the positive association of government guidelines. Nevertheless, they also observed that in contexts with heightened sociocultural restrictions or resistance, the outcomes were less promising, reflecting the idea that the mere presence of policies does not guarantee their success if social acceptance and effective resource availability are not achieved.

In Specific Hypothesis 3, addressing the relationship between public policies and maternal morbidity, the model exhibited a Chi-square change of 49.397 (*p* = 0.000), while Nagelkerke’s R^2^ stood at 0.562, indicating that 56.2% of the variation in maternal morbidity was attributable to public policies. With negative and statistically significant coefficients for low and medium policy levels, it became evident that weaker government initiatives were associated with more severe complications during adolescent pregnancy or childbirth.

These findings concur with those reported by [11, 12], and [30], who documented a rise in obstetric complications (such as preeclampsia, anemia, and preterm births) in the absence of adequate prenatal care and policies ensuring comprehensive coverage for pregnant adolescents. This study demonstrated that when public policies were ranked low or implemented deficiently, the probability of maternal morbidity rose, aligning with [12], who viewed sexual education and family planning as vital components for mitigating risk factors. Similarly [13], noted locally that specialized medical services are not uniformly available, limiting the effectiveness of interventions. Although none of these authors entirely dispute the necessity of public policies, they underscore the importance of adapting strategies to each region’s specific realities, recognizing the contextual differences that can influence the impact of such policies on maternal morbidity.

Lastly, Specific Hypothesis 4, which focused on maternal mortality in adolescents, revealed a Chi-square change of 52.282 (*p* = 0.000) and a Nagelkerke R^2^ of 0.578, indicating that about 57.8% of the variance in maternal mortality was related to public policies. As in earlier cases, the coefficients for low and medium levels of state intervention were negative and statistically significant.

These outcomes align with the arguments of [12] regarding the importance of preventive measures and training healthcare staff to identify and manage complications in a timely manner, as well as with [21], who stressed the need to expand obstetric services and remove geographical and socioeconomic barriers. In the same way [25], concurs that lacking a state structure prioritizing pregnant adolescents not only increases mortality risks but also perpetuates inequality cycles.

In short, both the consistencies and possible divergences with previous studies underscore that public policies are a key factor in mitigating adolescent pregnancy and its effects on maternal health. When supplemented by comprehensive sex education, qualified healthcare personnel, and effective access to services and contraceptive methods, government directives were perceived to be associated with lower pregnancy rates and related complications. Otherwise, their impact is limited by shortages of resources, insufficient awareness among healthcare professionals, or cultural and economic gaps that continue to restrict adolescents’ right to health.

### Cross-hypothesis synthesis

The ordinal-logit models show a clear gradient in explanatory power: early adolescent pregnancy exhibited the highest Nagelkerke R² (0.706), followed by late adolescent pregnancy (0.631), maternal mortality (0.578) and maternal morbidity (0.562). The magnitude of the χ² statistics mirrors this pattern (Table 6 vs. Tables 8, 10 and 12), indicating that public-policy implementation explains a larger share of variance in events occurring at younger ages.

Comprehensive sexuality-education policies, measured in the sexual-health domain, focus on delaying sexual initiation and improving condom use, two factors that directly affect pregnancies among 10- to 14-year-olds. In contrast, pregnancies at 15–19 years and obstetric complications depend not only on behavioural prevention but also on service availability, budgets and referral mechanisms; these elements are captured in the reproductive-health and inter-sectoral-coordination domains, which showed smaller but still significant effects.

The higher responsiveness of early pregnancy to policy implementation suggests that primary prevention (education, early counselling) may yield quicker, more measurable outcomes than secondary prevention (management of morbidity) or tertiary prevention (mortality reduction). This finding aligns with UNFPA reports that link early pregnancy declines to school retention and contraceptive uptake.

Strengthening budgeting and coordination could narrow the gap between early and late adolescent outcomes, while maintaining investment in sexuality education. Because our data are perception-based and cross-sectional, longitudinal monitoring with registry indicators is required to confirm whether the observed gradient persists over time.

### Limitations related to sampling

This study relied on convenience sampling, which may introduce selection bias and limits the external validity of the findings. Although the sample covered 79% of the obstetric workforce in the network, professionals who were unavailable on the survey days might hold different views. The high response rate mitigates, but does not eliminate, this concern. Future studies should employ probabilistic designs or multi-site approaches to improve external validity.

### External indicators and future work

Recent registry-based evidence supports our perception findings. A retrospective ecological study using Peru’s national birth-certificate registry reported an adolescent pregnancy rate of 0.28 per 100 live births among girls aged 10–14 years during 2017–2021, with higher rates in jungle regions [54]. That study also showed that greater secondary-school completion and contraceptive use were associated with lower pregnancy rates. These objective trends are consistent with the associations perceived by professionals in our survey. The research team is currently compiling longitudinal ENDES and MINSA service-coverage data to cross-validate perceived policy effectiveness with registry indicators in a follow-up analysis.

## Conclusions

Our results indicate three statistically significant associations: health networks with full implementation of comprehensive sexuality-education policies, guaranteed budget allocation for adolescent-friendly contraception services, and effective inter-sectoral coordination mechanisms were all associated with lower perceived early adolescent pregnancy, obstetric morbidity and maternal mortality (all *p* < 0.001). Because these findings derive from a cross-sectional survey of professional perceptions rather than registry data, causality cannot be inferred; nevertheless, the strength of the associations underscores the practical value of strengthening policy implementation to reduce adolescent-pregnancy-related risks in similar rural settings.

## Recommendations

Strengthen the effective implementation of public policies. Based on the evidence from this study, it is suggested to reinforce the execution of concrete, well-funded actions, as indicated by [28], who underscore those merely symbolic policies, lacking resources and clear procedures, limit their potential association with lower adolescent-pregnancy levels and preventing related complications. Therefore, it is recommended to create or consolidate management teams that oversee adherence to guidelines at the regional level and coordinate with local agencies to ensure sustainability.

Expand comprehensive sex education coverage from early ages. Findings on early adolescent pregnancy highlight the necessity of timely educational interventions, a view consistent with [30] and [26]. To accomplish this, it would be advisable to integrate curricular modules targeting both students and teaching staff, emphasizing access to contraceptive methods, sexual violence prevention, and bodily autonomy. It is also recommended to develop educational guides tailored to various school levels and to conduct periodic monitoring of their implementation.

Increase access to contraceptive methods and sexual and reproductive health counseling. In line with [31] and [27], it is advised to enhance the availability of contraceptive methods and personalized guidance, giving priority to areas with higher rates of adolescent pregnancy. Equally significant is the need to train healthcare personnel in differentiated care for adolescents, promoting an empathetic and non-discriminatory attitude, and establishing flexible schedules or adapted facilities that make it easier for this demographic to seek and receive care.

Implement prevention and control programs for maternal morbidity in adolescents. The results regarding maternal morbidity align with [30] and [12], who underscore the need for rigorous monitoring throughout pregnancy, childbirth, and the postpartum period. It is therefore proposed to consolidate a surveillance system that identifies risk factors, enhances prenatal education, and guarantees prompt access to medium- and high-complexity obstetric services. Additionally, it would be advantageous to establish effective referral and counter-referral protocols that ensure comprehensive care for adolescents and foster intersectoral coordination among health, education, and social protection.

Prioritize reducing maternal mortality through multisectoral strategies. In keeping with [25] and [21], it is suggested to design targeted policies aimed at decreasing pregnancy-related deaths among those under 19, emphasizing early detection of complications and access to emergency obstetric services. It would also be advisable to strengthen communication among healthcare facilities to ensure timely referral to higher-capacity centers without financial or geographical barriers. Involving both the community and family members in these initiatives could bolster adherence to prenatal care and enable prompt responses to warning signs.

## Supplementary Information

Supplementary Material 1.

Supplementary Material 2.

Supplementary Material 3.

Supplementary Material 4.

Supplementary Material 5.

Supplementary Material 6.

Supplementary Material 7.

## Data Availability

No datasets were generated or analysed during the current study.
