# Supplementary figures and images for: Public policies and their association with adolescent pregnancy in Southern Peru
Source: Reprod Health. 2025 Sep 30;22:172. doi: 10.1186/s12978-025-02131-w (PMC12486993; doi:10.1186/s12978-025-02131-w)

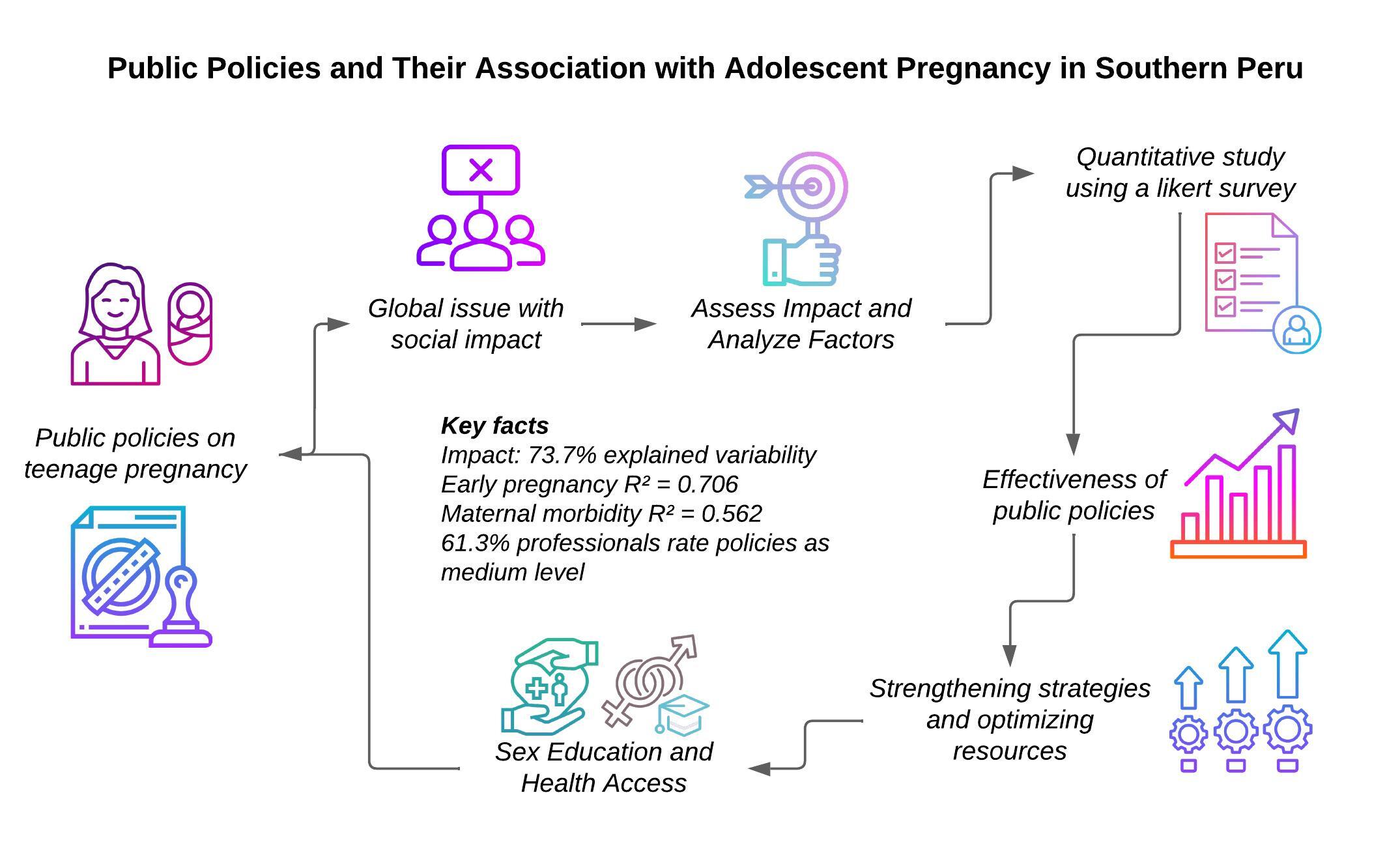

Supplement: Supplementary file 1 — Supplementary Material 1. [file 12978_2025_2131_MOESM1_ESM.jpeg]
