# Supplementary material for: Public policies and their association with adolescent pregnancy in Southern Peru
Source: Reprod Health. 2025 Sep 30;22:172. doi: 10.1186/s12978-025-02131-w (PMC12486993; doi:10.1186/s12978-025-02131-w)
Supplement: Supplementary file 2 — Supplementary Material 2. [file 12978_2025_2131_MOESM2_ESM.docx]

| Survey on Public-Policy Implementation and Its Association with  Adolescent Pregnancy in Health Networks  [ **SD –** Strongly disagree (1) / **D –** Disagree (2) / **N** = Neutral (3) / **A –** Agree (4) / **SA –** Strongly agree (5) ] | | | | | |
| --- | --- | --- | --- | --- | --- |
| ITEMS | SCALA | | | | |
| **Variable: Public policies** | SD | D | N | A | SA |
| **Dimension 1: Sexual health** | | | | | |
| 1. Public policies have helped delay early sexual initiation to prevent adolescent pregnancy. |  |  |  |  |  |
| 1. The Health Network implements strategies to delay early sexual initiation and prevent adolescent pregnancy |  |  |  |  |  |
| 1. Public policies have helped reduce sexual violence among adolescents to prevent adolescent pregnancy. |  |  |  |  |  |
| 1. The Health Network implements strategies to reduce sexual violence among adolescents and prevent adolescent pregnancy. |  |  |  |  |  |
| 1. Public policies have helped lower the incidence of sexually transmitted infections (STIs) in adolescents. |  |  |  |  |  |
| 1. The Health Network implements strategies to reduce sexually transmitted infections (STIs) among adolescents. |  |  |  |  |  |
| **Dimension 2: Reproductive health** | | | | | |
| 1. Public policies have improved access to family-planning services to prevent adolescent pregnancy. |  |  |  |  |  |
| 1. The Health Network implements strategies to improve access to family-planning services and prevent adolescent pregnancy. |  |  |  |  |  |
| 1. Facilities within the Health Network provide adolescent-friendly obstetric services as mandated by public policies. |  |  |  |  |  |
| 1. My workplace offers adolescent-friendly obstetric services in line with public-policy standards. |  |  |  |  |  |

| ITEMS | SCALA | | | | |
| --- | --- | --- | --- | --- | --- |
| **Variable: Adolescent pregnancy** | SD | D | N | A | SA |
| **Dimension 1: Early pregnancy** | | | | | |
| 1. Public policies have helped raise adolescents’ educational attainment, which is associated with lower early adolescent pregnancy. |  |  |  |  |  |
| 1. Public policies have helped reduce adolescent unions, thus helping to prevent early adolescent pregnancy. |  |  |  |  |  |
| 1. Public policies have helped lower poverty levels, which is associated with fewer early adolescent pregnancies. |  |  |  |  |  |
| 1. Public policies have improved communication between adolescents and their parents, contributing to lower early adolescent pregnancy. |  |  |  |  |  |
| 1. Public policies provide support for adolescents with absent parents, helping to prevent early adolescent pregnancy. |  |  |  |  |  |
| 1. Public policies address school dropout, thereby preventing early adolescent pregnancy. |  |  |  |  |  |
| 1. Public policies have helped decrease obstetric complications in early adolescent pregnancies. |  |  |  |  |  |
| **Dimension 2: Late pregnancy** | | | | | |
| 1. Public policies have helped raise adolescents’ educational attainment, which is associated with lower late adolescent pregnancy. |  |  |  |  |  |
| 1. Public policies have helped reduce adolescent unions, thus helping to prevent late adolescent pregnancy. |  |  |  |  |  |
| 1. Public policies have helped lower poverty levels, which is associated with fewer late adolescent pregnancies. |  |  |  |  |  |
| 1. Public policies have improved communication between adolescents and their parents, contributing to lower late adolescent pregnancy. |  |  |  |  |  |
| 1. Public policies provide support for adolescents with absent parents, helping to prevent late adolescent pregnancy. |  |  |  |  |  |
| 1. Public policies address school dropout, thereby preventing late adolescent pregnancy. |  |  |  |  |  |
| 1. Public policies have helped decrease obstetric complications in late adolescent pregnancies. |  |  |  |  |  |
| **Dimension 3 : Maternal morbidity** | | | | | |
| 1. Public policies have helped decrease maternal morbidity in early adolescent pregnancies. |  |  |  |  |  |
| 1. Public policies have helped decrease maternal morbidity in late adolescent pregnancies. |  |  |  |  |  |
| **Dimension 4: Maternal mortality** | | | | | |
| 1. Public policies have helped reduce maternal mortality in early adolescent pregnancies. |  |  |  |  |  |
| 1. Public policies have helped reduce maternal mortality in late adolescent pregnancies. |  |  |  |  |  |
