# Supplementary material for: Public policies and their association with adolescent pregnancy in Southern Peru
Source: Reprod Health. 2025 Sep 30;22:172. doi: 10.1186/s12978-025-02131-w (PMC12486993; doi:10.1186/s12978-025-02131-w)
Supplement: Supplementary file 3 — Supplementary Material 3. [file 12978_2025_2131_MOESM3_ESM.docx]

Encuesta sobre la Implementación de Políticas Públicas y su Asociación con el Embarazo Adolescente en Redes de Salud

| ITEMS | ESCALA | | | | |
| --- | --- | --- | --- | --- | --- |
| **Variable: Políticas Públicas** | MD | ED | I | DA | MA |
| **Dimensión 1: Salud sexual** | | | | | |
| 1. Las políticas públicas han logrado disminuir el inicio precoz de las relaciones sexuales para evitar el embarazo adolescente. |  |  |  |  |  |
| 1. En la Red Asistencial se aplican estrategias para la disminución del inicio precoz de las relaciones sexuales para evitar el embarazo adolescente |  |  |  |  |  |
| 1. Las políticas públicas han logrado reducir la violencia sexual en adolescentes para prevenir el embarazo adolescente. |  |  |  |  |  |
| 1. En la Red Asistencial se aplican estrategias para la reducción de la violencia sexual en adolescentes para prevenir el embarazo adolescente. |  |  |  |  |  |
| 1. Las políticas públicas han reducido la incidencia de infecciones de transmisión sexual (ITS) en adolescentes. |  |  |  |  |  |
| 1. En la Red Asistencial se aplican estrategias para reducir las infecciones de trasmisión sexual (ITS) en adolescentes. |  |  |  |  |  |
| **Dimensión 2: Salud reproductiva** | | | | | |
| 1. Las políticas públicas han mejorado el acceso a planificación familiar para evitar el embarazo adolescente. |  |  |  |  |  |
| 1. En la Red Asistencial se aplican estrategias para mejorar el acceso a planificación familiar para evitar el embarazo adolescente. |  |  |  |  |  |
| 1. Los establecimientos de salud de la Red Asistencial cuentan con servicios obstétricos diferenciados para adolescentes embarazadas como lo establecen las Políticas públicas. |  |  |  |  |  |
| 1. En el establecimiento que usted labora cuentan con servicios obstétricos diferenciados para adolescentes embarazadas como lo establecen las Políticas públicas |  |  |  |  |  |

| ITEMS | ESCALA | | | | |
| --- | --- | --- | --- | --- | --- |
| **Variable: Embarazo adolescente** | MD | ED | I | DA | MA |
| **Dimensión 1: Temprano** | | | | | |
| 1. Las políticas públicas han incrementado el nivel educativo de las adolescentes, reduciendo el embarazo adolescente temprano. |  |  |  |  |  |
| 1. Las políticas públicas han contribuido a disminuir las uniones de adolescentes, evitando el embarazo adolescente temprano. |  |  |  |  |  |
| 1. Las políticas públicas han reducido los niveles de pobreza, disminuyendo el embarazo adolescente temprano |  |  |  |  |  |
| 1. Las políticas públicas han mejorado la comunicación entre adolescentes y sus padres, reduciendo el embarazo adolescente temprano. |  |  |  |  |  |
| 1. Las políticas públicas intervienen en el apoyo a adolescentes cuyos padres están ausentes, evitando el embarazo adolescente temprano. |  |  |  |  |  |
| 1. Las políticas públicas combaten la deserción escolar, previniendo el embarazo adolescente temprano. |  |  |  |  |  |
| 1. Las políticas públicas han disminuido las complicaciones obstétricas en el embarazo adolescente temprano. |  |  |  |  |  |
| **Dimensión 2: Tardío** | | | | | |
| 1. Las políticas públicas han incrementado el nivel educativo de las adolescentes, reduciendo el embarazo adolescente tardío |  |  |  |  |  |
| 1. Las políticas públicas han contribuido a disminuir las uniones de adolescentes, evitando el embarazo adolescente tardío. |  |  |  |  |  |
| 1. Las políticas públicas han reducido los niveles de pobreza, disminuyendo el embarazo adolescente tardío |  |  |  |  |  |
| 1. Las políticas públicas han mejorado la comunicación entre adolescentes y sus padres, reduciendo el embarazo adolescente tardío. |  |  |  |  |  |
| 1. Las políticas públicas intervienen en el apoyo a adolescentes cuyos padres están ausentes, evitando el embarazo adolescente tardío. |  |  |  |  |  |
| 1. Las políticas públicas combaten la deserción escolar, previniendo el embarazo adolescente tardío. |  |  |  |  |  |
| 1. Las políticas públicas han disminuido las complicaciones obstétricas en el embarazo adolescente tardío. |  |  |  |  |  |
| **Dimensión 3: Morbilidad** | | | | | |
| 1. Las políticas públicas han reducido la morbilidad materna en el embarazo adolescente temprano |  |  |  |  |  |
| 1. Las políticas públicas han reducido la morbilidad materna en el embarazo adolescente tardío |  |  |  |  |  |
| **Dimensión 4: Mortalidad** | | | | | |
| 1. Las políticas públicas han reducido la mortalidad materna en un embarazo adolescente temprano |  |  |  |  |  |
| 1. Las políticas públicas han reducido la mortalidad materna en un embarazo adolescente tardío |  |  |  |  |  |
