# Supplementary material for: Public policies and their association with adolescent pregnancy in Southern Peru
Source: Reprod Health. 2025 Sep 30;22:172. doi: 10.1186/s12978-025-02131-w (PMC12486993; doi:10.1186/s12978-025-02131-w)
Supplement: Supplementary file 6 — Supplementary Material 6. [file 12978_2025_2131_MOESM6_ESM.docx]

Highlights

**Public Policies and Their Association with Adolescent Pregnancy in Southern Peru**

Emilio Medrano-Sánchez, Lizabeth Alanya- Pereyra, Freddy Ochoa-Tataje

- Social factors associated with pregnancy
- Survey showed high internal reliability (Cronbach α = 0.83)
- Policies explained 73.7 % of adolescent-pregnancy variance
- Early pregnancy most responsive to policy action (R² = 0.706)
- Sex-education, funded contraception and coordination all p < 0.001
- Findings inform SDG-aligned actions in rural health networks
